# Supplementary material for: Causal Relationship between Aging and Anorexia Nervosa: A White-Matter-Microstructure-Mediated Mendelian Randomization Analysis
Source: Biomedicines. 2024 Aug 16;12(8):1874. doi: 10.3390/biomedicines12081874 (PMC11351342; doi:10.3390/biomedicines12081874)
Supplement: Supplementary file 1 [file biomedicines-12-01874-s001.zip › Supplementary Figure/Figure S8/Leaveoneout plot of MR analysis for TL to ubm-b-1564.pdf]

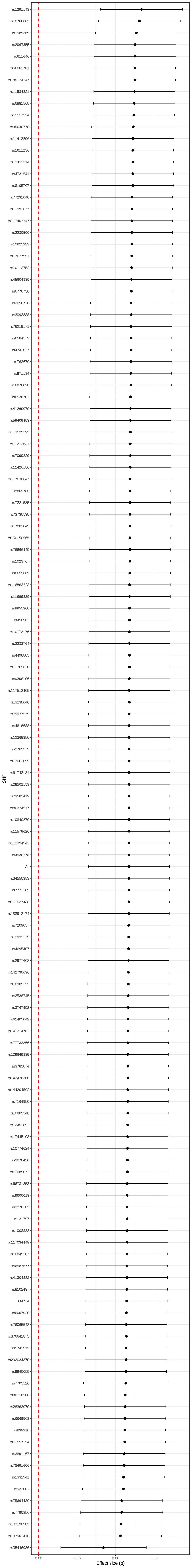
rs1291143
rs10768683
rs1985369
rs2967355
rs611646
rs56061761
rs185174247
rs11584821
rs6881568
rs11117354
rs35640778
rs11412296
rs1611236
rs12412214
rs4731541
rs8105767
rs77231040
rs11991877
rs117407747
rs2230590
rs12925933
rs17677991
rs10112752
rs45604339
rs6776756
rs2056726
rs3093888
rs76219171
rs6584579
rs4743037
rs762679
rs871134
rs16978028
rs6536702
rs41269079
rs59409453
rs113525195
rs11212631
rs7099229
rs11426156
rs117630647
rs869785
rs7221585
rs73730598
rs17803849
rs150150565
rs76666449
rs1023767
rs6659669
rs116863223
rs11699829
rs9955360
rs450962
rs10773176
rs2282764
rs4498805
rs11769630
rs9398196
rs117512405
rs13230646
rs79977579
rs4616688
rs12369950
rs2763979
rs13062095
rs61748181
rs28502153
rs73581419
rs80324517
rs10840270
rs11579626
rs112394943
rs4530278
All
rs34550383
rs7772289
rs111527438
rs188918174
rs7209057
rs12932179
rs4695407
rs2977608
rs142730696
rs10905255
rs2538745
rs3767952
rs61405042
rs141214782
rs77732866
rs139669835
rs3785074
rs142426306
rs144204502
rs7164950
rs10805346
rs12451892
rs17445108
rs10774624
rs9878436
rs11085072
rs66731853
rs9600019
rs2276182
rs131797
rs1003322
rs117034449
rs10845387
rs6587577
rs41304832
rs8102497
rs4724
rs6007020
rs76065543
rs376641875
rs5742915
rs202034370
rs9940099
rs7705526
rs80116508
rs28363070
rs6669563
rs939916
rs11557154
rs3891167
rs78491606
rs1332941
rs932002
rs75664430
rs7790856
rs143190905
rs137901416
rs35446936
SNP
0.00
0.03
0.06
0.09
Effect size (b)
